# Qualitative research literacy among pediatric oncology providers: institutional capacity, barriers, and implications for patient-centered cancer care in a middle-income setting

**DOI:** 10.3389/fonc.2026.1871228

**Published:** 2026-07-29

**Authors:** Rawad Rihani, Nisrein Al-Aqqad, Iyad Sultan, Maha Barbar, Khawlah Ammar, Haneen Abaza, Anwar Al Rifai, Majeda A. Al-Ruzzieh, Omar Shamieh, Tharwa Bilbeisi, Asem Mansour

**Affiliations:** 1 Department of Pediatrics, King Hussein Cancer Center., Amman, Jordan; 2 School of Medicine, the University of Jordan., Amman, Jordan; 3 Artificial Intelligence and Data Innovation Office, King Hussein Cancer Center., Amman, Jordan; 4 Office of Scientific Affairs and Research, King Hussein Cancer Center, Amman, Jordan; 5 Department of Nursing, King Hussein Cancer Center., Amman, Jordan; 6 Center for Palliative & Cancer Care in Conflict (CPCCC), Department of Palliative Care, King Hussein Cancer Center, Amman, Jordan; 7 Department of Global Pediatric Medicine, St. Jude Children’s Research Hospital, Memphis, TN, United States; 8 Office of the Director General, King Hussein Cancer Center, Amman, Jordan

**Keywords:** barriers, capacity building, knowledge and experiences, pediatric oncology, qualitative research, survey, training needs

## Abstract

**Background:**

Qualitative research plays a critical role in pediatric oncology by highlighting patient and caregiver experiences, psychosocial challenges, and contextual barriers to care. The ability of oncology teams to interpret and engage with qualitative evidence may influence the integration of patient-centered insights into clinical practice and service improvement. However, institutional capacity for qualitative research engagement among interdisciplinary pediatric oncology providers in low- and middle-income settings remains underexplored.

**Methods:**

We conducted a cross-sectional survey of healthcare providers working in the Department of Pediatrics at a comprehensive cancer center in Jordan. A structured 29-item questionnaire was used to assess prior exposure to qualitative research, knowledge of qualitative concepts, perceived barriers to participation, confidence levels, and preferred training methods. Descriptive and inferential analyses were conducted to explore associations between knowledge, training exposure, and professional characteristics.

**Results:**

A total of 137 providers participated, mainly nurses (74.4%) and female staff (83.2%), mirroring the workforce composition. While 98.4% recognized the importance of qualitative research in pediatric oncology, objective knowledge scores revealed significant gaps (mean score 39.8%). Providers with postgraduate education and prior research experience had significantly higher knowledge levels (p<0.001). Frequently reported barriers to engagement included limited time (58.4%) and inadequate training (55.4%). Despite these challenges, 96.6% expressed interest in future involvement with qualitative research, preferred structured workshops (64.2%), and online courses (36.5%).

**Conclusion:**

Although pediatric oncology providers acknowledged the value of qualitative research in understanding patient and family experiences, institutional capacity to engage with and apply qualitative methodologies remains limited. Targeted educational initiatives and structured mentorship models could enhance qualitative research literacy and improve the integration of patient-centered insights into oncology care delivery, particularly in resource-limited settings.

## 1 Introduction

Pediatric oncology care extends beyond biomedical treatment to include complex psychosocial, emotional, cultural, and systemic dimensions that significantly influence patient and family experiences. Qualitative research provides an in-depth understanding of these lived experiences and offers insights that quantitative methodologies alone cannot capture (1, 2). In pediatric settings, qualitative approaches have been crucial for highlighting caregiver distress, communication challenges, survivorship experiences, and the social determinants that affect treatment trajectories (3–6). By integrating social, emotional, and contextual dimensions into healthcare evaluation, qualitative research contributes to patient-centered care, service redesign, and policy development (2, 5). Despite its recognized value, qualitative research remains underutilized in the health sciences relative to quantitative methodologies (7, 8). This imbalance may limit the oncology team’s ability to systematically incorporate patient narratives and contextual insights into care improvement initiatives.

In low- and middle-income countries (LMICs), research capacity development faces additional structural barriers, including limited funding, constrained institutional infrastructure, insufficient methodological training, and weak integration between research and policy implementation (9–12). These challenges may further limit both the production and effective application of qualitative research in oncology settings. Previous studies have assessed research knowledge and attitudes among medical researchers (13) and health sciences faculty members (14), revealing gaps in methodological familiarity and confidence in research. However, evidence remains limited on qualitative research literacy and engagement among interdisciplinary pediatric oncology providers, particularly in LMIC cancer centers.

In this study, qualitative research literacy refers to healthcare providers’ foundational familiarity with qualitative research approaches and their relevance to clinical practice. This includes a basic understanding of qualitative methodologies, the ability to interpret qualitative findings in literature, and the capacity to recognize how evidence derived from qualitative studies can inform patient care, service development, and research collaboration. Importantly, qualitative research literacy does not imply that all pediatric oncology providers must independently conduct qualitative studies. Rather, it reflects the level of awareness and understanding that enables interdisciplinary healthcare professionals to engage meaningfully in qualitative research, collaborate effectively with trained qualitative researchers, and integrate patient and family perspectives into oncology practice.

To address this gap, our study aimed to assess healthcare providers’ knowledge of qualitative research, prior exposure to qualitative research, perceived barriers, and training preferences within the Department of Pediatrics at King Hussein Cancer Center (KHCC). By mapping institutional qualitative research capacity in a middle-income pediatric oncology setting, we sought to identify opportunities to strengthen engagement with patient-centered qualitative evidence in cancer care.

## 2 Methods

### 2.1 Study design

This cross-sectional study was conducted at the Department of Pediatrics at King Hussein Cancer Center (KHCC), Amman, Jordan, between July and October 2024. The study is reported in accordance with the STROBE (Strengthening the Reporting of Observational Studies in Epidemiology) guidelines for cross-sectional studies (**Supplementary File 1**).

A survey-based design was selected to enable broad institutional assessment of qualitative research knowledge, exposure, and perceived barriers across multiple professional roles within the pediatric oncology workforce. Given the objective of mapping institutional-level qualitative research literacy and engagement readiness rather than exploring individual experiences in depth, a quantitative survey approach was considered appropriate.

### 2.2 Study setting

KHCC is a comprehensive center of excellence in the Middle East serving approximately 7,000 new cancer patients annually, including approximately 500 pediatric patients (15, 16). Survival rates for certain pediatric cancers exceed 85% (17). KHCC maintains active international collaborations addressing regional challenges in cancer diagnosis, treatment, and survivorship (18). The Department of Pediatrics operates through multidisciplinary collaboration involving physicians, nurses, psychosocial professionals, pharmacists, and nutritionists.

### 2.3 Study participants and sampling method

All healthcare providers working within or directly collaborating with the Department of Pediatrics were eligible to participate, including pediatric oncology consultants, specialists, fellows, residents, nurses, pharmacists, psychologists, social workers, and nutritionists.

The total eligible population was approximately 250 providers. A census-based recruitment strategy was employed, inviting all eligible staff to participate. An initial minimum target of 152 responses was estimated using conventional survey sampling assumptions for institutional surveys (19).

### 2.4 Instrument development

A structured online questionnaire comprising 29 questions in English was developed. This format followed the working language proficiency of healthcare providers at KHCC. Questions focused on participants’ prior experience, knowledge, and perceived barriers related to conducting qualitative research, as well as their preferred methods for further learning about the topic. The five knowledge questions assessed key concepts related to qualitative research: 1. qualitative sample size determination, 2. qualitative research approaches, 3. qualitative interview formats, 4. focus group size, and 5. qualitative data analysis software. All items except three were presented in a multiple-choice format, and some questions allowed multiple responses. (**Supplementary File 2**).

The survey was developed through a sequential process. First, the research team conducted an extensive literature review to identify the main domains from previous studies. Seven questions were adapted from prior studies and surveys on research knowledge and practices (14, 20–30). Qualitative research experts refined some questions to ensure alignment with qualitative research methodologies. The question addressing barriers to conducting qualitative research was developed after a review of the literature on broader research challenges and was tailored to reflect issues specific to qualitative inquiry (20–27). Questions exploring prior research training and experience were adapted from previous studies, with modifications to contextualize them within qualitative research (23, 28–30). In addition, four questions assessing knowledge of qualitative methodologies were derived from a previously published qualitative research study (14).

The draft instrument underwent iterative internal review by the principal investigator and research specialists at KHCC to assess clarity, relevance, and alignment with qualitative research constructs. An internal expert review process was conducted to refine the instrument. Faculty members with research experience evaluated the clarity of items and their conceptual alignment with qualitative research literacy domains. Based on feedback, wording was adjusted to improve precision and reduce ambiguity. Formal content validity indices were not calculated, which represents a methodological limitation.

#### 2.4.1 Pilot testing

The questionnaire was piloted with 11 pediatric oncology consultants to assess clarity, comprehension, and interpretability. Participants provided structured feedback. Based on this pilot, three items (education level, professional role, and years of experience) were revised to improve completeness of response categories and clarity. Although pilot feedback improved clarity, no formal re-evaluation of measurement properties was conducted after revisions.

### 2.5 Data collection

The refined questionnaire was structured into seven main sections: Sociodemographic Information, Training in Qualitative Research, Preferences and Learning Methods, Barriers to Conducting Qualitative Research, Confidence and Readiness, Future Interest and Engagement, and Previous Knowledge of Qualitative Research. The survey was accompanied by a cover letter explaining the aims, voluntary participation, and expected duration of the study.

The survey was administered electronically using SurveyMonkey^®^. The survey link was distributed through departmental chairpersons and managers to ensure broad dissemination across professional roles. Quick-response (QR) codes were displayed on departmental flyers to facilitate convenient access. Data collection occurred over three months to accommodate varying clinical schedules and maximize participation.

### 2.6 Data analysis

Statistical analysis was performed using Statistical Package for the Social Sciences (SPSS) version 29. Descriptive statistics included frequencies and percentages for categorical variables and means with standard deviations for continuous variables. Inferential statistical analysis was performed to examine relationships between variables and compare group differences. These analyses included one-way ANOVA to compare means across multiple groups, independent-samples t-tests for two-group comparisons, Chi-square tests for associations between categorical variables, and Pearson correlation tests for relationships between continuous variables. A *p*-value of less than 0.05 was considered significant.

Knowledge scores were calculated by summing correct responses to knowledge-based questions and converting the total into a percentage (0-100%).

### 2.7 Ethics approval and consent to participate

Ethical approval was obtained from the Institutional Review Board (IRB) at King Hussein Cancer Center (KHCC) in June 2024, IRB number 24 KHCC 106. Informed consent was obtained from all participants before participation via a cover page that preceded the survey. The study was conducted in compliance with the Jordanian Clinical Research Law (2011), which adheres to the principles of the Declaration of Helsinki, and in accordance with Good Clinical Practice (GCP) guidelines.

## 3 Results

### 3.1 Participant characteristics

A total of 137 healthcare providers completed the survey. Given the descriptive and exploratory nature of this study, the achieved sample size was considered sufficient to characterize institutional qualitative research literacy, although subgroup comparisons may be underpowered.

Participants had a mean age of 30.6 years (range 21–57). The majority were female (83.2%) and nurses (74.4%), consistent with the workforce composition of the Pediatric Department at KHCC. Most participants held bachelor’s degrees (77.3%), followed by master’s degrees (19%). Mean professional experience was 6.4 years (range <1–22 years). (**Table 1**).

**Table 1:** Demographic characteristics of study participants.

Characteristic	(N = 137) n (%)
Age (years), mean (range)	30.6 (21–57)
Gender	
Female	114 (83.2%)
Male	23 (16.8%)
Highest education level	
Bachelor’s degree (including residents)	106 (77.3%)
Master’s degree (including specialists, fellows)	26 (19%)
PhD (including consultants)	1 (0.7%)
Secondary certificate (High school diploma)	1 (0.7%)
Clinical role	
Physicians	15 (10.9)
Nurses	102 (74.4%)
Allied health professionals	11 (8%)
Pharmacists	9 (6.6%)
Years of experience	
Less than 5 years	62 (45.3%)
5–10 years	50 (36.5%)
More than 10 years	25 (18.2%)

### 3.2 Training and research experience

The level of qualitative research training varied among participants. Almost a third had never received any training in qualitative research, while a slightly larger percentage received limited training, primarily through lectures; a smaller group had received only moderate training through workshops and short courses; and only a few participants had undergone extensive training through long courses or one-to-one training. (**Table 2**) There were no significant differences in previous qualitative research training across clinical roles (χ^2^, *p* = 0.814).

**Table 2:** Prior qualitative research training among healthcare providers.

Level of qualitative research training	n (%)
No training	45 (32.8%)
Limited training (primarily lectures)	51 (37.2%)
Moderate training (workshops, courses)	30 (21.9%)
Extensive training	11 (8.0%)

Overall, 40.9% of participants had prior research experience of any type. Among those, 31.5% participated in qualitative research, while others reported quantitative or mixed-methods involvement. Participants’ primary roles in qualitative research included serving as principal investigators (5.8%), identifying potential participants (5.8%), approaching participants (3.6%), performing data analysis/coding (3.6%), developing data collection tools (2.9%), conducting data collection (2.9%), transcription (1.5%), and translation (0.7%).

More female healthcare workers participated in research than males (44.5% vs. 22.7%); however, this was not statistically significant (*p* = 0.062). Although participants with research training were more likely to conduct research (46.6% vs. 29.5%), and specifically, qualitative research (43.9% vs. 25%), these differences were not statistically significant (*p* = 0.064 and *p* = 0.323, respectively).

### 3.3 Knowledge assessment and confidence

Although 98.4% of participants acknowledged the importance of qualitative research in pediatric oncology, 54.6% self-rated their knowledge as fair, and 19.3% reported having good knowledge, objective knowledge assessment revealed substantial gaps. The mean knowledge score was 39.8%. Most respondents (90.7%) provided incorrect responses to the data analysis question (qualitative data analysis software).

Participants with postgraduate education demonstrated significantly higher knowledge scores compared to those with undergraduate education (65.8% vs 32.9%, *p* < 0.001). Similarly, participants with prior research experience had significantly higher knowledge scores than those without research experience (49% vs 32.9%, *p* < 0.001). Likewise, participants who participated in qualitative research training had a higher level of knowledge compared to those who did not (42.6% vs. 33.5%), although the results were not statistically significant (*p* = 0.057). (**Table 3**).

**Table 3:** Comparison of mean knowledge score by receipt of qualitative research training.

Training in qualitative research	N	Mean knowledge score	Std. deviation	p-value
Had any kind of training	82	42.68	31.07	0.057
None	37	33.51	20.03	

Most participants expressed modest confidence in conducting qualitative research in pediatric oncology. Among participants, 27% expressed extreme confidence, 59% reported fair confidence, and 13.9% lacked confidence.

### 3.4 Challenges to qualitative research engagement

**Figure 1:**
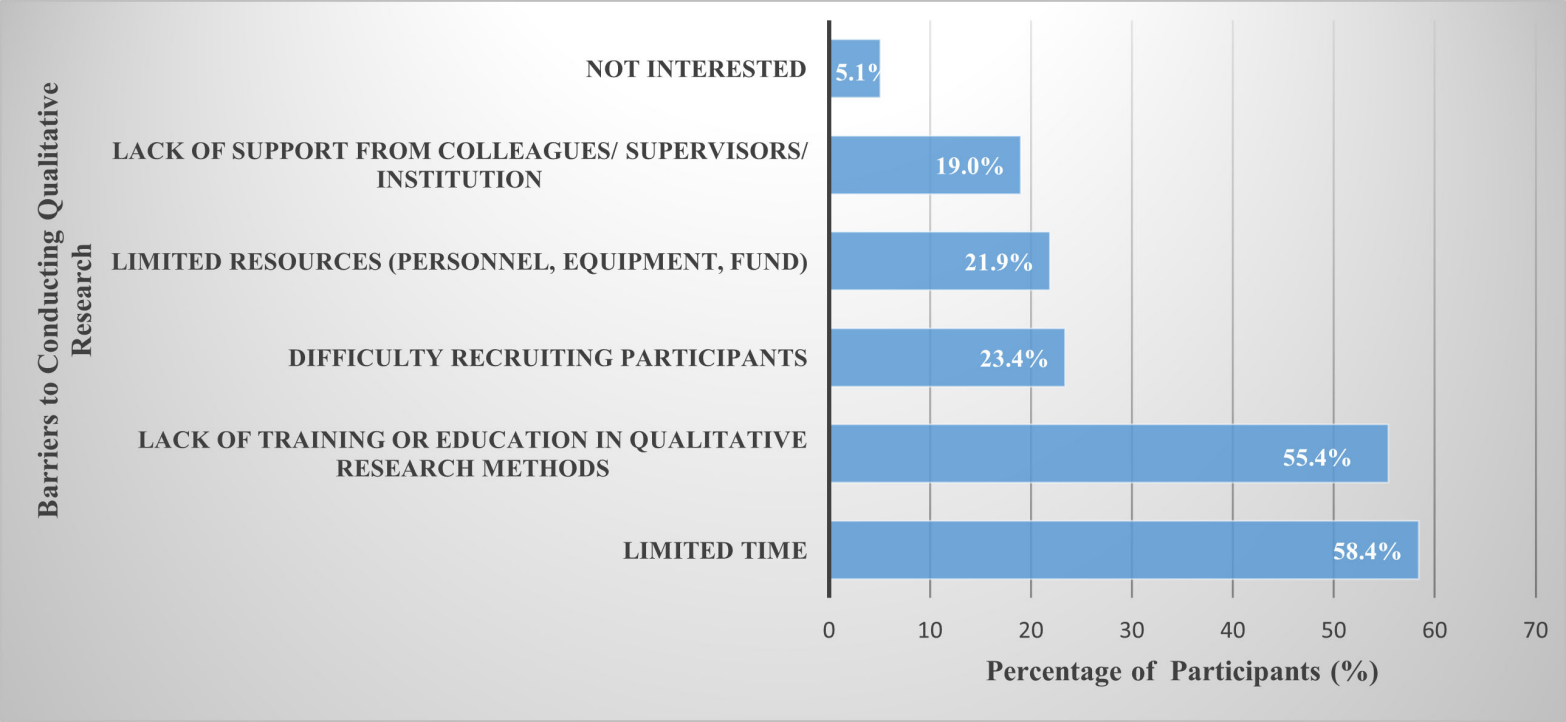
Barriers to conducting qualitative research reported by healthcare providers in the department of pediatrics at King Hussein Cancer Center.

The most frequently reported barriers to engagement in qualitative research included organizational barriers such as lack of time, insufficient training, difficulty recruiting participants, and limited resources. (**Figure 1**) Notably, 93.3% of physicians considered a lack of training as a serious challenge, compared to 51.5% of nurses.

The most reported difficulty involved data analysis and coding (41.6%). This was closely followed by idea generation/conceptualization (40.9%) and creating data collection tools like interview guides (37.2%). Other difficulties encountered by participants included conducting interviews or focus group discussions (27%), finding potential participants (16.8%), transcription (15.3%), and translation (14.6%).

### 3.5 Learning preferences and future engagement

**Figure 2:**
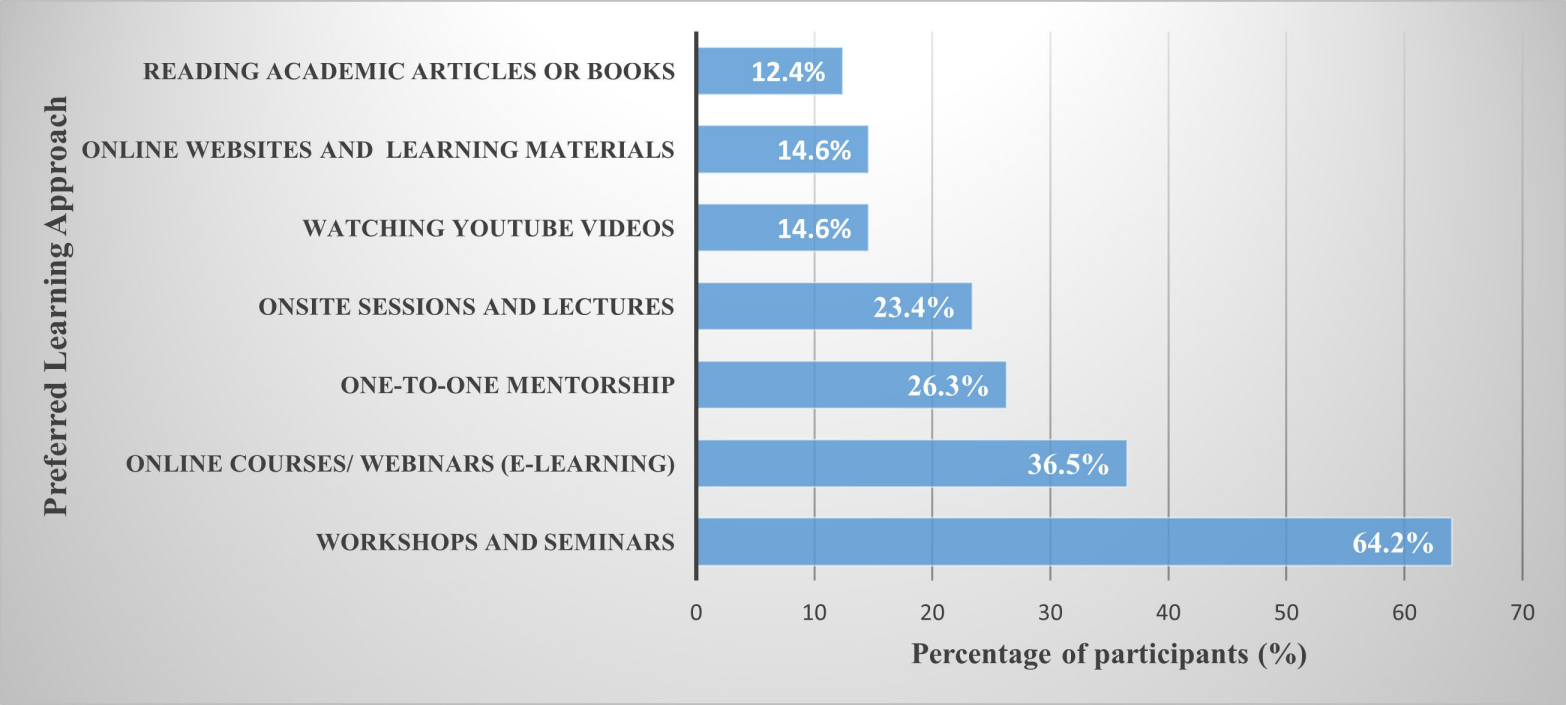
Preferred learning methods for qualitative research in pediatric oncology reported by healthcare providers working with the department of pediatrics at King Hussein Cancer Center.

Despite knowledge gaps, 96.6% of participants expressed interest in future engagement with qualitative research, subject to the availability of time, training opportunities, and resources. We found that healthcare providers with higher education levels were slightly more interested in conducting qualitative research than those with lower education levels (80% vs. 66%). Still, this difference was not statistically significant (*p* = 0.315). Preferred learning modalities included: workshops and seminars, online courses or webinars, and mentorship-based learning (**Figure 2**).

## 4 Discussion

This study offers one of the first assessments of interdisciplinary qualitative research literacy and engagement readiness within a pediatric oncology workforce in a middle-income setting. It provides an overview of the workforce’s preparedness to adopt qualitative research. This was conducted through a comprehensive evaluation of previous knowledge, experience, perceived barriers, and training preferences.

Nearly all participants acknowledged the importance of qualitative research in understanding patient and caregiver experiences, and most showed interest in future participation. However, objective knowledge assessment revealed substantial gaps in knowledge and barriers to participation. These findings align with prior studies demonstrating limited methodological familiarity among health faculty members and medical researchers (13, 14).

A key finding is the gap between perceived and actual knowledge. Most participants (73.9%) rated their knowledge as “fair” or good, yet the mean knowledge score was only 39.8%. This discrepancy may reflect overconfidence or under-recognition of the need for foundational training. It may also reflect that some knowledge questions required more than basic understanding and assessed methodological concepts. This has implications for tailored capacity-building programs, as healthcare providers who overestimate their knowledge may be less motivated to engage in training or seek methodological support.

The low performance on data analysis questions (9.3%) may reflect both limited methodological exposure and the technical complexity of this domain. While this suggests a training priority, the difficulty level of this specific item should be interpreted cautiously, as it requires hands-on experience with qualitative data analysis software (e.g., NVivo and MAXQDA), which many participants may not have been exposed to or used before.

Qualitative research contributes uniquely to pediatric oncology. It captures emotional, social, and contextual dimensions of illness that are often not measurable through quantitative approaches (1, 3, 4). Such insights are particularly relevant in pediatric cancer care, where communication, psychosocial support, adherence, and survivorship challenges shape outcomes (31). Despite this relevance, qualitative methods remain underrepresented in health services and management research compared with quantitative designs (7, 8). This imbalance is also evident within our institution, where research activity has predominantly focused on quantitative designs, underscoring the need to assess and strengthen qualitative research capacity.

Our findings suggest that even within oncology settings where patient-centered care is prioritized, familiarity with qualitative methods may remain limited. Importantly, this study does not suggest that all healthcare providers should independently conduct qualitative research. Rather, interdisciplinary collaboration is central to pediatric oncology research and to improving care. A foundational understanding of qualitative methodologies may facilitate meaningful engagement in collaborative research, improve interpretation of qualitative findings, and support translation of patient-experience insights into service development initiatives. This collaboration involves multidisciplinary teams of clinicians and allied health professionals working with researchers through joint study design, iterative interpretation of qualitative data, and shared dissemination of findings. Such collaboration can integrate different disciplinary perspectives and improve the use of qualitative evidence in clinical decision-making and patient-centered service development within pediatric oncology care. This is supported by previous research showing that qualitative insights from caregivers can directly inform psychosocial oncology practice (32).

Consistent with prior literature examining research barriers in LMICs, participants reported structural constraints, including limited time, insufficient training, and resource limitations (9–12). Similar barriers have been described in studies assessing research engagement among healthcare professionals in various contexts (20, 22, 24–26).

Participants with postgraduate education and prior research experience demonstrated significantly higher knowledge scores, consistent with earlier findings linking academic exposure to research literacy (14). No significant differences were observed across clinical roles in previous qualitative research training, but this should be interpreted with caution given the small sample size (n = 11) of allied health professionals, which limited subgroup comparisons and the ability to detect potential differences between professional groups. Moreover, psychosocial roles in LMIC oncology settings often operate within clinical rather than research frameworks, which may explain similar training levels.

The strong interest in future qualitative research engagement, despite current knowledge gaps, indicates a receptive workforce for structured capacity-building initiatives.

Preferred learning modalities, including workshops and online courses, provide practical direction for developing tiered educational programs that accommodate varying levels of engagement. However, selection of educational modality should also be guided by the specific learning objectives and the likelihood of achieving meaningful educational impact, rather than preferences alone.

In pediatric oncology settings within LMICs, strengthening qualitative research literacy may support culturally responsive care, enhance communication strategies, and inform context-specific interventions (31). Given documented systemic barriers to research development in LMIC contexts, targeted institutional strategies may help bridge gaps between patient experience insights and oncology service delivery (9–12).

From an implementation science perspective, these findings support integrating qualitative research training into institutional capacity-building and continuing professional development programs. They also highlight the need to embed qualitative methods in routine research and quality improvement to support knowledge translation into practice. In addition, the results reinforce the importance of patient-centered care models that incorporate patient and caregiver experiences into clinical decision-making and service design.

### 4.1 Strengths and limitations

A key strength of this study is that it is among the first to examine qualitative research literacy across an interdisciplinary healthcare team in the pediatric oncology in a middle-income setting. By examining knowledge levels, prior exposure, perceived barriers, and preferred learning approaches together, we gained a broad view of the healthcare providers’ readiness to engage more meaningfully with qualitative research.

Several limitations could be identified. The single-institution design limits generalizability beyond comparable oncology centers. Although the response rate was substantial relative to the eligible population, subgroup analyses may be underpowered. Additionally, the predominance of nurses in the workforce reflects the composition but may also influence aggregate knowledge estimates. The survey-based design captures perceived barriers and knowledge but does not explore contextual factors in depth. This limits the ability to fully understand how organizational culture, leadership support, workload pressures, and prior knowledge and training shape engagement with qualitative research. Future qualitative studies could further elucidate how institutional dynamics influence research engagement. In addition, the survey items were not formally validated and may not fully capture the most relevant competencies for collaborative qualitative research. Some items may reflect more specialized methodological knowledge rather than practical ability to engage in interdisciplinary research, which should be considered when interpreting the findings. This may affect the accuracy of measuring participants’ knowledge and should be considered when interpreting the knowledge score.

### 4.2 Conclusion

While interdisciplinary providers showed considerable interest in qualitative research and recognized its value in pediatric oncology, this study reveals gaps in knowledge and preparedness to engage with qualitative methods within a pediatric oncology workforce in a middle-income context. Constraints such as limited familiarity with qualitative methodologies and organizational barriers may hinder effective participation and the interpretation of qualitative findings.

Rather than advocating universal methodological expertise, our findings underscore the value of strengthening qualitative research literacy through tiered and context-sensitive training strategies. Such institutional capacity-building efforts may enhance collaborative research engagement and support the integration of patient and caregiver perspectives into pediatric oncology service development. In LMIC contexts, strengthening qualitative research engagement may contribute to more contextually responsive and patient-centered oncology care.

## Data Availability

The original contributions presented in the study are included in the article/**Supplementary Material**. Further inquiries can be directed to the corresponding author.
