# Supplementary material for: Qualitative research literacy among pediatric oncology providers: institutional capacity, barriers, and implications for patient-centered cancer care in a middle-income setting
Source: Front Oncol. 2026 Jul 29;16:1871228. doi: 10.3389/fonc.2026.1871228 (PMC13461546; doi:10.3389/fonc.2026.1871228)
Supplement: Supplementary file 2 [file Table2.docx]

***Qualitative Research Literacy Among Pediatric Oncology Providers: Institutional Capacity, Barriers, and Implications for Patient-Centered Cancer Care in a Middle-Income Setting***

Section 1: Sociodemographic Information

Top of Form

* 1. What is your gender:

Male

Female

* 2. How old are you (in years)

* 3. What is your highest educational level

Diploma

Bachelor Degree (including residents)

Master’s Degree (including specialists, fellows)

PhD (including consultants)

Other (please specify)

* 4. What is your role in pediatric oncology?

Pediatric oncology consultant

Pediatric consultant (non oncology)

Pediatric specialist

Pediatric oncology fellow

Pediatric resident

Nurse

Nurse Coordinator

Psychologist

Social worker

Nutritionist

Pharmacist

Other (please specify)

* 5. How many years of experience do you have in pediatrics?

Section 2: Training in Qualitative Research

Top of Form

* 6. Have you **received** any formal training or education in qualitative research methods previously?

Yes, extensively (e.g. long courses, one-to-one training )

Yes, moderately (e.g. short courses, workshops)

Yes, limited (e.g. lectures)

No, none at all

7. If you have received training in qualitative research methods, please indicate the source (s) of your training or knowledge. (Select all that apply)

University or academic institution

Workshops or seminars

Online courses or webinars

Mentorship from colleagues or supervisors

Other (please specify)

8. Have you ever conducted **research** either as a PI or co-investigator?

Yes

No

9. If you answered “Yes” to the previous question, please identify the research design: (Select all that apply)

Quantitative Research

Qualitative Research

Mixed-methods Research

I don’t know

 10. Have you ever conducted **qualitative** research as a PI or co-investigator?

Yes

No

11. If you answered "Yes" to the previous question, please identify your role in the qualitative research (select all that apply):

Principal Investigator

Identifying potential participants

Approaching potential participants

Developing interview/Focus Group guide

Conducting interviews/Focus Group Discussions/Observation

Transcription

Translation

Data analysis/coding

Other (please specify)

12.What methods do you **prefer** for learning about qualitative research? (Select all that apply)

Workshops or seminars (group training)

Online courses or webinars (E-learning)

Reading academic articles or books

One-to-one training or mentorship

Watching YouTube videos

Onsite sessions and lectures

Online website and learning materials

Other (please specify)

* 13. What is important to you when you choose a learning opportunity? (select all that apply)

It offers a certificate

Affordable or free to access

It is provided by a recognized organization

Flexible completion timeframe

Relevant to current work

Other (please specify)

* 14. How important do you think it is for pediatric healthcare providers to have knowledge of qualitative research?

Very important

Moderately important

Not important

Section 4: Barriers to Conducting Qualitative Research

Top of Form

* 15. Which in your opinion is difficult about qualitative research stages/steps? (Select all that apply)

Idea generation (conceptualization)

Identifying potential participants

Approaching potential participants

Designing data collection tools (interview guide)

Conducting interviews/focus group discussions

Transcription

Translation

Data analysis/ coding

Other (please specify)

* 16. What do you perceive as **barriers** to conducting qualitative research? (Select all that apply)

Lack of training or education in qualitative research methods

Limited time

Difficulty recruiting participants

Lack of support from colleagues/ supervisors/ institution

I am not interested

Limited resources (personnel, equipment, fund)

No barriers

Other (please specify)

Section 5: Confidence and Readiness

Top of Form

* 17. How confident do you feel in your ability to **conduct** a qualitative research study in pediatric oncology?

Very confident

Moderately confident

Not confident at all

Bottom of Form

18. What factors contributed to your confidence in conducting qualitative research? (Select all that apply)

Previous training or education in qualitative research methods

Experience conducting qualitative research studies

Support from colleagues/ supervisors or institution

Access to resources such as funding/ equipment/ personnel

Other (please specify)

 19. What factors contributed to your lack of confidence in conducting qualitative research? (Select all that apply)

No previous training or education in qualitative research methods

Lack of experience conducting qualitative research studies

Lack of support from colleagues/ supervisors or institution

Lack of access to resources such as funding/ equipment/ personnel

Other (please specify)

Section 6: Future Interest and Engagement

Top of Form

20. Would you be interested in participating in **future** qualitative research projects? (Select all that apply)

Yes

Maybe

No

21. If you answered "Maybe" then depends on what

Depending on the research topic

If I received training

If I have time and resources

- Other (please specify)

* 22. What types of qualitative research topics or areas would you be interested in exploring within pediatric oncology? (Select all that apply)

Patient experiences and perspectives

Caregiver experiences and challenges

Healthcare provider perspectives and practices

Quality of life and psychosocial outcomes

Other (please specify)

Section 7: Previous Knowledge of Qualitative Research

Top of Form

* 23. How would you rate your knowledge of qualitative research?

Good

Fair

Poor

* 24. A guiding principle in deciding the sample size in qualitative research is:

Number of variables

Effect size

**Data saturation**

Sub-group Analysis

* 25. A qualitative approach that focuses on the description and interpretation of cultural behavior is:

**Ethnography**

Narrative

Case study

Phenomenology

* 26. Which data collection method would you use when you have no pre-determined list of questions:

Semi-structured interviews

**Unstructured interview**

Multiple interview

Structured interview

* 27. The recommended size of typical focus group discussion is: (Choose the best answer)

11-15

2-3

**6-10**

20 and above

* 28. Which of the following software are used for qualitative data analysis: (Select all that apply)

SPSS

STATA

**MAXQDA**

**Nvivo**

All of the above

None of the above

#### 29. Suggest titles for qualitative research at the department of Pediatrics

Top of Form

Bottom of Form

Bottom of Form
